# Burr Hole Washout versus Craniotomy for Chronic Subdural Hematoma: Patient Outcome and Cost Analysis

**DOI:** 10.1371/journal.pone.0115085

**Published:** 2015-01-22

**Authors:** Jacqueline M. Regan, Emmagene Worley, Christopher Shelburne, Ranjit Pullarkat, Joseph C. Watson

**Affiliations:** 1 University of Michigan, Ann Arbor, MI, United States of America; 2 Virginia Commonwealth University, Inova Campus, Falls Church, VA, United States of America; 3 Inova Fairfax Hospital, Department of Neurosciences, Falls Church, VA, United States of America; 4 Cerebrum MD, Vienna, VA, United States of America; City of Hope, UNITED STATES

## Abstract

Chronic subdural hematomas (CSDH), which are frequently encountered in neurosurgical practice, are, in the majority of cases, ideally treated with surgical drainage. Despite this common practice, there is still controversy surrounding the best surgical procedure. With lack of clear evidence of a superior technique, surgeons are free to base the decision on other factors that are not related to patient care. A retrospective chart review of 119 patients requiring surgical drainage of CSDH was conducted at a large tertiary care center over a three-year period. Of the cases reviewed, 58 patients underwent craniotomy, while 61 patients underwent burr hole washout. The study focused on re-operation rates, mortality, and morbidity, as measured by Glasgow coma scores (GCS), discharge Rankin disability scores, and discharge disposition. Secondary endpoints included length of stay and cost of procedure. Burr hole washout was superior to craniotomy with respect to patient outcome, length of stay and recurrence rates. In both study groups, patients required additional surgical procedures (6.6% of burr hole patients and 24.1% of craniotomy patients) (*P* = 0.0156). Of the patients treated with craniotomy, 51.7% were discharged home, whereas 65.6% of the burr hole patients were discharged home. Patients who underwent burr hole washout spent a mean of 78.8 minutes in the operating suite while the patients undergoing craniotomy spent 129.4 minutes (*P* < 0.001). The difference in mean cost per patient, based solely on operating time, was $2,828 (*P* < 0.001). This does not include the further cost due to additional procedures and hospital stay. The mean length of stay after surgical intervention was 3 days longer for the craniotomy group (*P* = 0.0465). Based on this retrospective study, burr hole washout is superior for both patients’ clinical and financial outcome; however, prospective long-term multicenter clinical studies are required to verify these findings.

## Introduction

Chronic subdural hematoma (CSDH), an increasingly common neurosurgical disease, is readily diagnosed on non-contrast CT (computed tomography) scans. The reported incidence is approximately 3 per 100,000 and rises appreciably in the elderly population [1]. Though there is a consensus that surgical drainage is the preferred treatment for symptomatic patients, the ideal surgical approach remains controversial [2].

The most common procedures for treatment of CSDH include twist drill craniostomy, single or multiple burr hole drainage, and craniotomy [3]. Recent articles state that burr hole drainage is a superior technique compared to twist drill craniostomy and craniotomy, due to a lower incidence of recurrence and morbidity [3, 4]. However, no Class I data comparing these treatments head to head[3] has been established to eliminate the debate over the optimal surgical approach. In consequence, the choice of surgical procedure is influenced by factors other than clinical outcome. Surgeons choosing to perform craniotomy charge more than those choosing to perform burr hole washout, though preferring a procedure based on revenue is a potential conflict of interest.

At our institution the two surgical drainage procedures presently being performed to treat CSDH are burr hole washout and “mini” craniotomy. Despite burr hole washout being the most common procedure reportedly performed around the world [2, 4–8][3, 9], at our institution the numbers of patients receiving treatment via bur hole washout was roughly equal to those who were treated using the mini craniotomy technique.

Proponents for performing a craniotomy argue that the wide exposure allows for loculations to be broken up and membranes to be opened, which in turn leads to increased amount of subdural drained and decreased recurrence rate [10, 11]. The purpose of this study was to compare the procedures, determining if there was indeed a lower recurrence rate or other exceeding clinical benefits that would justify the large number of craniotomies performed at our institution.

## Methods

After Inova Human Research Protection Program Institutional Review Board study approval (study #10.147), the charts of all adult patients requiring surgical drainage of a chronic subdural hematoma from January 1, 2007 until December 31, 2009 were retrospectively reviewed. Due to the retrospective nature of this study, no consent was given and all records were de-identified in compliance with the IRB. One hundred and twenty-three patients were identified with four patients being excluded for the following reasons: underlying aneurysm, active leukemia, or functioning ventriculoperitoneal shunt.

The primary endpoints of the study included re-operation rates and mortality. Secondary endpoints involved length of post-operative stay and morbidity as measured by post-operative and discharge Glasgow coma score, discharge Rankin disability scores, and discharge disposition. Estimated cost of procedure was also recorded. The data collection concluded when the patient was either discharged or expired. There was no long-term follow-up.

To analyze primary and secondary endpoints, mean values were compared using 2 sample t-tests. Comparison of proportions was performed using the Z test. All tests were two sided and calculated using IBM’s SPSS statistical analysis software (SPSS, Inc., v. 19, 2010, Armonk, NY). In all circumstances a probability (*P*) value of <0.05 was considered statistically significant.

All patients underwent either burr hole drainage, via one, two, or in one case three burr holes, or a “mini-craniotomy”, which is defined as a craniotomy of 5–7 cm in greatest diameter. The choice of operation was based solely on the surgeon’s preference and clinical experience. Evidence of multiple loculations and age of the patients had no influence on the surgical technique as none of the surgeons strayed from their normal method of treating chronic subdural hematomas. The patient population in our retrospective study is consistent with published reports of typical demographics representative of CSDH patients [1, 5, 7, 8, 10, 12](Table 1).

**Table 1:** Comparison of patient demographics.

	**Burr Hole Group**	**Craniotomy Group**	**P-value**
**Age** (mean)	72	68	0.1158
**Gender** (% male)	59	67	0.4599
**Initial GCS** (mean)	14.5	13.6	0.2660
**Rankin disability score** (mean)	2.7	2.6	0.7284

Cranial non-contrast CT scans were completed using the same imaging protocol, which consisted of a 512×512 matrix with 5 mm axial slices. An initial CT scan was performed on arrival and a follow up CT scan was done within 24 hours post procedure. Clot volume of subdural hematoma was measured using the formula AxBxC/2, where A, B and C represent the dimensions in three axes perpendicular to each other [13]. The amount of hemorrhage on the pre and post-operative CT scans were calculated and recorded. The difference was then computed and the percentage removed determined. Medical records were reviewed for basic patient demographics; preoperative, postoperative day 1 and discharge Glasgow coma score; admission and discharge Rankin disability score; pre-operative co-morbidities; use of anticoagulation/antiplatelet therapy; history of alcohol abuse; use of subdural drain; time in the operating room as well as associated cost with operating room use; and appearance and size of the hematoma on both pre-operative and post-operative imaging studies.

The surgical technique for the “mini-craniotomy” involved raising a craniotomy flap of about 5–7 cm in greatest diameter centered over the area of maximal hematoma thickness. The outer membrane was opened and excised as far as the craniotomy would allow and then the inner membrane was opened and coagulated as well. The burr hole washouts consisted of two burr holes in the majority of the cases in which a burr hole was placed frontal at Kocher’s point and the other placed at the parietal boss. The outer membrane was opened and irrigation was performed until clear. The vast majority, 77 of the 80 burr hole patients and 61 of the 80 craniotomy patients, had intra-operative placement of a subdural drain.

## Results

Over the three-year period at a large tertiary care center 119 patients underwent 160 CSDH procedures by 10 neurosurgeons. The craniotomy group was comprised of 58 patients who had 80 craniotomies. Seven required bilateral craniotomy, 14 patients returned to the OR, and one patient required two additional operations. The 61 patients undergoing burr hole washout also required a total of 80 procedures as 13 patients underwent bilateral drainage and four patients were taken back to the operating room with two of them requiring re-drainage of both sides. Of patients undergoing burr hole washout, 70 (87.5%) underwent placement of two burr holes, nine (11.25%) had drainage via one burr hole, and the remaining one case received three burr holes with the additional over the temporal area.

The age and sex were similar in both groups (Table 1). The mean age was 72 years in the burr hole group (range 52–98 y) and 68 years in the craniotomy group (range 37–89 y). The majority of the patients were male in both groups (59% and 67% in the burr hole and craniotomy groups respectively). The etiology was secondary to remote trauma for 62.3% of patients that underwent burr hole washout and 72.3% that received craniotomies. Twenty-nine patients in the burr hole group and 12 patients in the craniotomy group had a history of being on anticoagulation/anti-platelet therapy (*P* < 0.05). Platelet function assays were not performed on all patients but all patients on clopidogrel received platelets prior to any surgical intervention. Patients on warfarin had their international normalized ratio corrected to <1.6 for those undergoing burr hole washout and <1.3 for those receiving craniotomy prior to surgical procedure. Nine burr hole washout patients and seven craniotomy patients had a history of alcohol abuse. Initial mean Glasgow coma score and Rankin disability scores were found to be very similar (Table 1) with only the Glasgow coma score on POD #1 showing a statistically significant difference with a mean of 14.14 and 12.96 in the burr hole and craniotomy groups respectively (*P* = 0.0088). The location of the CSDH was on the right side in 49 patients, left sided in 48 patients and bilateral in the remaining 20 patients. The average thickness of the hematoma as seen on the pre-operative CT scan was similar, 18.4 mm in those having burr hole washout vs. 19.8 mm those receiving craniotomy (*P* = 0.1145).

The re-operation rates displayed a noticeable difference when comparing the groups (*P* = 0.0156) (Table 2). Re-operation was primarily due to residual CSDH without symptomatic improvement or secondary to acute hemorrhage(s). A total of only four patients (6.6%) that underwent burr hole washout were required return to the operating room for re-evacuation of symptomatic residual CSDH or acute hemorrhage caused by the original surgery. Of these four patients, two needed to be converted into craniotomies and the others required re-opening and irrigation of original burr holes. Of the patients that underwent craniotomy as treatment of CSDH, 14 (24.1%) required re-operation for the same reasons. One of those patients required two additional operations. In the majority of cases a subdural drain was placed; however, three patients (3.75%) in the burr hole group and 19 patients (23.5%) in the craniotomy group did not receive a drain (*P* < 0.001). Of the patients that were taken back for re-operation, four (100%) of the burr hole patients and 11 (79%) of the craniotomy patients had had subdural drain placed at the time of their original surgery.

**Table 2:** Primary and secondary endpoint results.

	**Burr Hole**	**Craniotomy**	**P-value**
Reoperation rates	6.6%	24.1%	0.0156
Length of post-operative stay (d)	7.3	10.3	0.0465
Operative time (min)	78.8	129.4	<0.001
Operative cost ($)	$7,588	$10,416	<0.001

**Fig 1:**
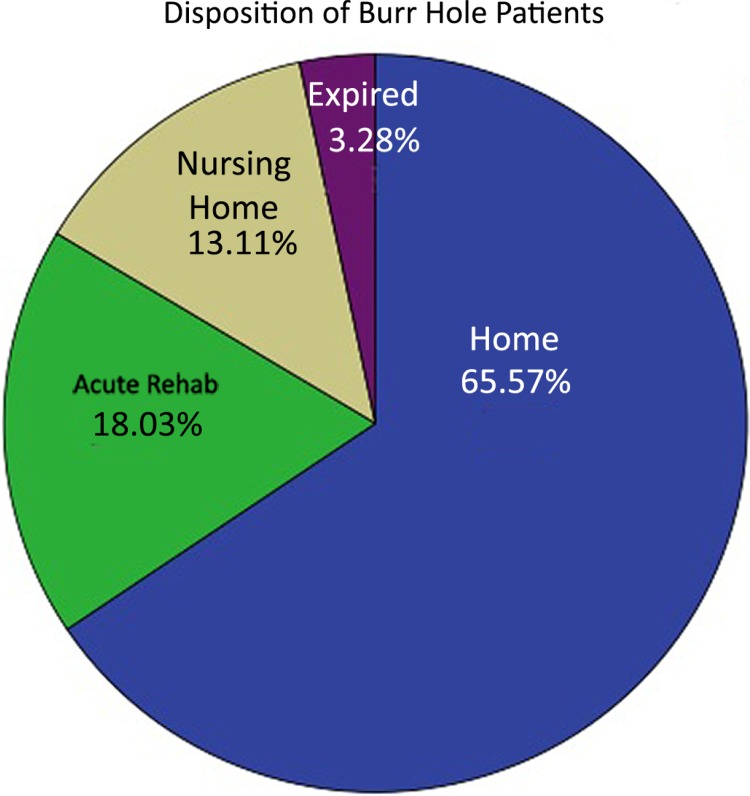
Burr hole group discharge disposition. Pie chart representing the disposition after discharge from the hospital for the burr hole group.

**Fig 2:**
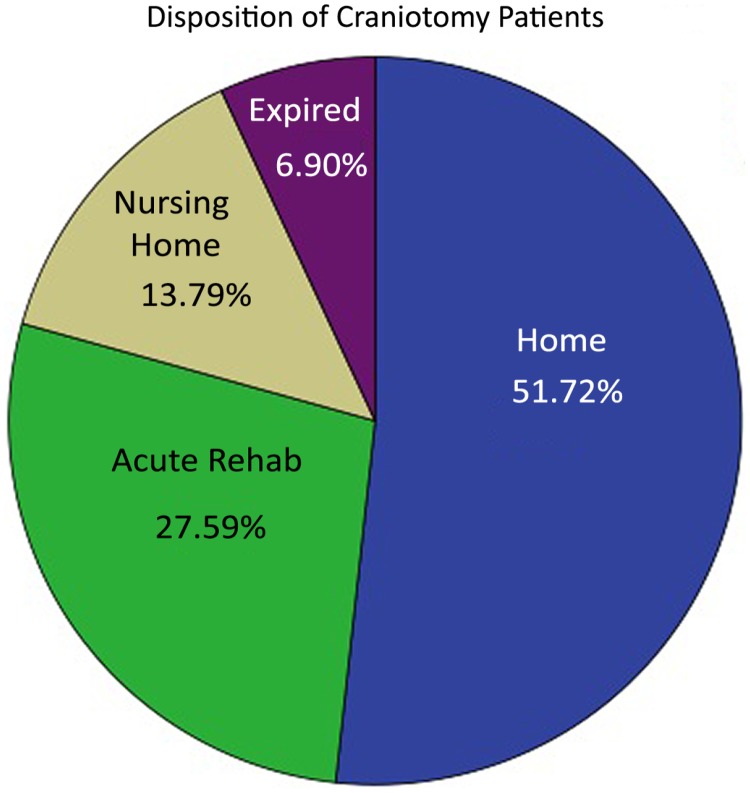
Craniotomy group discharge disposition. Pie chart representing the disposition after discharge from the hospital for the craniotomy group.

Discharge Glasgow coma score, Rankin disability scores as well as the mortality rates were similar in the two groups. Of the patients that underwent burr hole washout, 65.6% were discharged to home, 13.1% required a skilled nursing facility, 18% went to inpatient rehab and 3.3% expired (two patients)(Fig. 1). The mortalities in this group were due to brainstem infarction and to acute mechanical valve thrombosis as this patient was taken off warfarin and given fresh frozen plasma for an international normalized ratio of <1.3. Of those that were surgically treated with craniotomy, 51.7% were discharged home, 13.8% required placement into a skilled nursing facility, 27.6% proceeded to inpatient rehabilitation, and 6.9% expired (4 patients)(Fig. 2). The mortalities were caused by one case of sepsis and three families opting to withdraw care due to poor clinical prognosis.

There was greater incidence of post-operative complications in the craniotomy study group with a total of 32 events (55.2%) (*P* < 0.001). These consisted of clinical seizures (3), acute stroke (4), new onset arrhythmia (1), acute intracranial hemorrhages (10), deep vein thrombosis/ pulmonary embolism (1), myocardial infarction (1), respiratory failure (3) and post-operative infections (8), which included pneumonia (4), uncomplicated urinary tract infections (3) and one superficial wound infection. This compared to only 13 patients that received burr hole washout who suffered post-operative complications (21.3%), which included seizures (1), acute stroke (2), new onset arrhythmia (1), acute intracranial hemorrhages (2), deep vein thrombosis/pulmonary embolism (2), myocardial infarction (1), respiratory failure (1) and post operative infections (3). The postoperative infections consisted of two uncomplicated urinary tract infections and one case of cellulitis due to line placement.

Though the volume of hematoma and percentage removed on the POD #1 CT were similar; 47% in the burr hole group and 40% in the craniotomy group, the time spent in the operating room proved to be significantly longer for those undergoing craniotomy {average operating room time 78.8 and 129.4 minutes for the burr hole washout and craniotomy respectively (*P* < 0.001)} (Table 2). The mean cost per patient, based solely on operating time, was $7,588 for the burr holes patients and $10,416 for the craniotomy patients. Due to the dissimilarity in operation length, the difference in mean cost for use of the operating room alone was $2,828 (*P* < 0.001) (Table 2). Post-procedure length of stay proved longer for the craniotomy patients with a mean of 7.3 and 10.3 days for the burr hole and craniotomy groups respectively (*P* = 0.0465) (Table 2).

## Discussion

Chronic subdural hematoma is a common disease entity encountered in neurosurgical practice. The most common surgical treatments, twist drill craniostomy, burr hole drainage, and craniotomy, result in differing degrees of re-operation rates from 5% to 27.8% [5, 8] [7, 14] as well as varying morbidity and mortality rates. However, due to the discrepancy in reported recurrence rates of each surgical intervention there is controversy regarding the optimal surgical treatment. Mondorf et. al. describes a comparison between craniotomy and burr hole treatments where the number of craniotomy patients more than triples that of the burr hole group [12]. Sambasivan compares 2,300 cases of CSDH where over 2,200 are treated with craniotomy and only 51 with burr hole drainage [7]. Lee et al. compares 38 patients with burr hole drainage to 13 treated with craniotomy [8]. The difference in surgical treatment numbers correlates to skewed results and uncertain interpretation, thus making the choice of optimal surgical treatment more difficult to ascertain. To reevaluate disagreements between reported data, this study analyzes 61 patients treated with burr hole washout and 58 with craniotomy focusing on endpoints of re-operation, morbidity, and mortality rates.

The re-operation rate for CSDH treated with burr hole washout was 6.6% (four patients out of 61), which proved a significant difference from the 24.1% (14 of 58) of craniotomy patients requiring re-operation. The decreased number of required reoperations with burr hole washout made treatment with burr hole superior to craniotomy in our patient population. Additionally, there were a higher number of postoperative complications for craniotomy in our data, specifically related to postoperative infections, acute hemorrhage, and acute stroke. Mortality associated with both procedures was comparable. GCS and Disability Rankin Scale were used to assess patient functional outcomes. While difference between groups did not reach statistical significance at discharge, patients treated with burr hole washout were more likely to be discharged home. With similar morbidity and mortality rates, but reduced reoperation rates, our data support burr hole washout over minicraniotomy for treatment of CSDH.

Often, drains are employed to reduce the risk of hematoma recurrence and thus reoperation. Data from Weigal et. al. supports the use of closed system drainage in the reducing the risk of hematoma recurrence[3] and thus the need for re-operation. Santarius et. al. randomized 215 patients with CSDH treated with burr hole washout into two groups: drain vs. no drain. Those with drain were found to have a lower re-operation rate due to decreased recurrence of the hematoma [15]. Mori and Maeda reported a re-operation rate of 9.8% in 500 patients with burr hole craniotomy with closed system drainage [7]. Mondorf et. al., reported 14.3% re-operation in 42 patients [12]. Similarly, in this study, 95% of the patients treated with burr hole washout and 67% of the craniotomy group underwent closed system drainage post-operatively. There is controversy over how essential a subdural drain is in treatment of CSDH; however, lack of subdural drain placed in original surgery was not the overarching trigger for re-operation and instead the surgery method itself. An interesting comparison to data from the pediatric population (age < 2 years) shows patients with burr hole treatment required more subsequent procedures when compared to patients undergoing minicraniotomy [16]. In our data, the average age of the patients, 72 and 68 years of age in the burr hole and craniotomy groups respectively, cannot speak for the most effective treatment of chronic subdural hematomas in pediatric populations.

Secondary endpoints of the study, the average length of stay, average time in the operating room, and average cost of treatment were compared. The results support burr hole washout as superior to that of craniotomy in that there was shorter length of stay (8.7 vs. 11.1 days), less time in the operating room (78.8 vs. 129.4 minutes) and less overall cost of procedure ($7,588 vs. $10,416). As health care costs continue to be a societal concern, surgeons who elect more expensive procedures for treatment of diseases such as chronic subdural hematoma, when less costly and more effective procedures are available, will find themselves under close scrutiny.

The limitations of our study are primarily related to the retrospective nature and the relatively small number of patients. Due to the retrospective nature, there is a lack of long-term follow-up in our study. As it may be difficult to generalize the conclusions from the smaller sample size, the difference in patient safety and cost require further investigation. Future long-term multi-institutional, prospective studies are needed to fully demarcate the differential outcomes due to procedure choice.

## Conclusions

Abovementioned controversy over the optimal surgical procedure for treatment of chronic subdural hematoma due to lack of evidence in the literature may lead to surgical decisions based not exclusively on patient outcome. Many patients continue to undergo craniotomy for the treatment of CSDH, but given the results of this study, burr hole washout appears to be superior to craniotomy for the treatment of CSDH with respect to patient outcome, re-operative rates, and length of stay. From a cost perspective, craniotomy patients incurred higher cost than burr hole patients due to longer operating time, higher number of additional procedures, and longer length of hospital stay. Though surgeons may elect procedures on a case-by-case basis and data analyzing surgeons’ process of procedure selection should be studied, there may be a possible conflict of interest for surgeons continuing to regularly perform craniotomies instead of burr hole washouts for chronic subdural hematoma treatment. Burr hole washout with a closed drainage system appears to be a superior first line treatment of CSDH for adult patients, but future long-term prospective, multi-center studies are needed.
